# Statement of Retraction

**DOI:** 10.1080/21655979.2026.2614883

**Published:** 2026-01-19

We, the Editor-in-Chief and Publisher of *Bioengineered (the “**Journal**”)*, have retracted the following articles that were published in the Special Issue titled “***ABUD-2019****”*, guest edited by Sunita Varjani, Duu-Jong Lee & Quanguo Zhang. *Bioengineered, 11(S13), originally published in 11(01)*.

- Varjani, S., Lee, D. J., & Zhang, Q. (2020). Valorizing agricultural biomass for sustainable development: biological engineering aspects. *Bioengineered, 11*(SI3), 522–523. https://doi.org/10.1080/21655979.2020.1759185
- Li, P., He, C., Li, G., Ding, P., Lan, M., Gao, Z., & Jiao, Y. (2020). Biological pretreatment of corn straw for enhancing degradation efficiency and biogas production. *Bioengineered, 11*(SI3), 251–260. https://doi.org/10.1080/21655979.2020.1733733
- Guo, S., Lu, C., Wang, K., Wang, J., Zhang, Z., Jing, Y., & Zhang, Q. (2020). Enhancement of pH values stability and photo-fermentation biohydrogen production by phosphate buffer. *Bioengineered, 11*(SI3), 291–300. https://doi.org/10.1080/21655979.2020.1736239
- Hu, K., Jia, S. Q., Yang, C., Sun, X., Chen, W., Wang, W., & Han, F. (2020). Combined freezing-thawing pretreatment and microbial electrolysis cell for enhancement of highly concentrated organics degradation from dewatered sludge. *Bioengineered, 11*(SI3), 301–310. https://doi.org/10.1080/21655979.2020.1736735
- Lan, M., Li, W., Chang, C., Liu, L., Li, P., Pan, X., … Jiao, Y. (2020). Enhancement on enzymolysis of pigskin with ultrasonic assistance. *Bioengineered, 11*(SI3), 397–407. https://doi.org/10.1080/21655979.2020.1736736
- Jing, Y., Li, F., Li, Y., Jin, P., Zhu, S., He, C., … Zhang, Q. (2020). Statistical optimization of simultaneous saccharification fermentative hydrogen production from corn stover. *Bioengineered, 11*(SI3), 428–438. https://doi.org/10.1080/21655979.2020.1739405
- Jansson, A. T., Patinvoh, R. J., Taherzadeh, M. J., & Horváth, I. S. (2020). Effect of organic compounds on dry anaerobic digestion of food and paper industry wastes. *Bioengineered, 11*(SI3), 502–509. https://doi.org/10.1080/21655979.2020.1752594

Following publication, the Publisher identified concerns regarding the editorial handling of the Special Issue and the peer review process.

An investigation by the Taylor & Francis Publishing Ethics & Integrity team in full cooperation with the Editor-in-Chief concluded that the articles included in this Special Issue were not peer-reviewed appropriately, in line with the Journal’s peer review standards and policy.

As the stringency of the peer review process is core to the integrity of the publication process, the Editor-in-Chief and Publisher have decided to retract all the articles within the above-named Special Issue. The Editor-in-Chief and the Publisher have not confirmed if all the authors were aware of the concerns with the peer review process.

The Journal is committed to correcting the scientific record and will fully cooperate with any institutional investigations into this matter. The authors have been informed of this decision.

We have been informed in our decision-making by our editorial policies and the COPE guidelines.

The retracted articles will remain online to maintain the scholarly record, but they will be digitally watermarked on each page as ‘Retracted’.

